# SSR and AFLP based genetic diversity of soybean germplasm differing in photoperiod sensitivity

**DOI:** 10.1590/S1415-47572010005000024

**Published:** 2010-06-01

**Authors:** Ram K. Singh, Virendra S. Bhatia, K. V. Bhat, Trilochan Mohapatra, Nagendra K. Singh, Kailash C. Bansal, K. R. Koundal

**Affiliations:** 1 National Research Centre for Soybean, IndoreIndia; 2 National Research Centre for DNA Fingerprinting, National Bureau of Plant Genetic Resources, Pusa Campus, New DelhiIndia; 3 National Research Centre on Plant Biotechnology, Indian Agricultural Research Institute, Pusa Campus, New DelhiIndia

**Keywords:** photoperiod response, SSR, AFLP, genetic diversity, soybean

## Abstract

Forty-four soybean genotypes with different photoperiod response were selected after screening of 1000 soybean accessions under artificial condition and were profiled using 40 SSR and 5 AFLP primer pairs. The average polymorphism information content (PIC) for SSR and AFLP marker systems was 0.507 and 0.120, respectively. Clustering of genotypes was done using UPGMA method for SSR and AFLP and correlation was 0.337 and 0.504, respectively. Mantel's correlation coefficients between Jaccard's similarity coefficient and the cophenetic values were fairly high in both the marker systems (SSR = 0.924; AFLP = 0.958) indicating very good fit for the clustering pattern. UPGMA based cluster analysis classified soybean genotypes into four major groups with fairly moderate bootstrap support. These major clusters corresponded with the photoperiod response and place of origin. The results indicate that the photoperiod insensitive genotypes, 11/2/1939 (EC 325097) and MACS 330 would be better choice for broadening the genetic base of soybean for this trait.

The photoperiod response is a major criterion, which determines the latitudinal adaptation of a soybean variety (Hartwig and Kiihl, 1979). A considerable variation in the relative sensitivity of soybean genotypes to differences in photoperiod has been reported (Sinclair and Hinson, 1992). Roberts *et al.* (1996) had also emphasized the importance of photoperiod-insensitivity in the improvement of soybean crop after characterizing soybean genotypes in conjunction with an analysis of the world-wide range of photo-thermal environments in which soybean crops are grown. Most of the Indian soybean cultivars (> 95%) were found to be highly sensitive to photoperiod that limits their cultivation in only localized area (Bhatia *et al.*, 2003). Thus, it is important to identify genetically diverse source of photoperiod-insensitivity gene(s) to broaden the genetic base of Indian soybean cultivars.

Better knowledge of the genetic similarity of breeding materials could help to maintain genetic diversity and sustain long-term selection gains. Furthermore, monitoring the genetic variability within the gene pool of elite breeding material could make crop improvement more efficient by the directed accumulation of favored alleles thus decreasing the amount of material to be screened. Several studies have used molecular markers to help in identification of genetically diverse genotypes to use in crosses in cultivar improvement programme. These studies have more success than conventional selection programme in producing productive lines from plant introduction/exotic lines crosses with elite lines (Maughan *et al.*, 1996; Thompson and Nelson, 1998). Among the molecular markers simple sequence repeats (SSR) are reproducible, co-dominant and distributed through out the genome. The AFLPs being dominant markers allow studying many loci simultaneously and generating highly reproducible markers that are also considered to be locus specific within a species (Maughan *et al.*, 1996). These two markers can detect higher levels of genetic diversity in soybean and have been utilized for many purposes including genome mapping, gene tagging, estimation of genetic diversity and varietal identification (Maughan *et al.*, 1995, 1996; Powell *et al.*, 1996; Cregan *et al.*, 1999; Brown-Guedira *et al.*, 2000; Narvel *et al.*, 2000; Ude *et al.*, 2003; Wange *et al.*, 2006; Singh *et al.*, 2008). However, no information is available on assessment of genetic diversity in response to photoperiodism in soybean. The present study was conducted to identify genetic diversity in the soybean gene pool for photoperiod insensitivity using SSR and AFLP markers.

One thousand soybean genotypes obtained from India, USA, Hungary, Philippines and Taiwan were screened for sensitivity to photoperiodism as described by Singh *et al.* (2008). Out of these 44 genotypes, 15 genotypes showing different degree of photoperiod insensitivity and 29 sensitive genotypes were selected for analysis using SSR and AFLP markers. The place of origin, EC number and their response to photoperiodism are given in Table 1. Ten leaves, one each from ten plants of 44 soybean genotypes were collected and DNA was isolated by the method described by Doyle and Doyle (1990).

Simple sequence repeat (SSR)/ microsatellite analysis was carried out using 40 mapped markers distributed on all the 20 chromosomes (Cregan *et al.*, 1999) (Table 2). Amplification was carried out in a 10 μL reaction mixture consisting of 1X PCR assay buffer (Bangalore Genei Pvt. Ltd., India), 200 μM of the four dNTPs (MBI Fermentas, Lithuania, USA), 12 ng (1.8 picomole) each of forward and reverse primers (Life Technologies, USA), 0.5 units of Taq DNA polymerase (Bangalore Genei Pvt. Ltd., India) and 25 ng template DNA. PCR reactions were carried out in a thermal cycler (Gene Amp 9600 model, version 2.01 from Perkin Elmer, USA) using the following cycling parameters: initial denaturation at 94 °C for 5 min, followed by 35 cycles of 94 °C for 1 min, 55 °C for 1 min, 72 °C for 2 min and finally a primer extension cycle of 7 min at 72 °C. The amplification products were separated on 3% metaphor agarose gels containing 1.5% gel star (FMC Bio Products, Rockland, USA). Gels were run for 3 h at 50 V in 1X TBE buffer. DNA fragments were visualized under UV light and photographed using a Polaroid photographic system. The size of the fragments was estimated using a 50-bp DNA ladder (MBI Fermentas, Lithuania).

AFLP fingerprints were generated based on the protocol of Zabeau and Vos (1993) with the AFLP Analysis System II (Invitrogen Corporation, Grand Island, NY) following the manufacturer's instructions. The size of the fragments was estimated using a 20-bp DNA ladder (MBI Fermentas, Lithuania).

The scoring of bands was done as present (1) or absent (0) for each AFLP and SSR marker allele and data was entered in a binary data matrix as discrete variables. Jaccard's coefficient of similarity was calculated and a dendrogram was constructed by using Unweighted Pair Group Method of Arithmetic Mean (UPGMA). The computer package NTSYS-PC Version 2.02 (Rohlf, 1998) was used for cluster analysis. The same software was used to perform the Mantel test of correlation between the cophenetic values and the Jaccard similarity coefficients to ascertain reliability of the obtained clusters. Robustness of the clustering pattern was also tested using bootstrap analysis using Free Tree - Free ware software (Pavlicek *et al.*, 1999). The polymorphism information content (PIC) was calculated for SSR marker as 1 - Σ *p*_*ij*_^2^ where *p*_*ij*_ is the frequency of the *j*^th^ allele of *i*^th^ marker (Weir, 1990) while PIC for AFLP marker was calculated as described by Powell *et al.* (1996).

Among the 40 SSR primer pairs used in the present study, 34 (85.0%) were polymorphic, while six primers revealed monomorphic patterns. In total, 120 alleles were detected for the 34 polymorphic SSR primers, with an average of 3.53 alleles per locus. Allele sizes ranged from 90 bp to 300 bp. Summarized data for the SSR loci and their PIC values are presented in Table 2. The PIC value, a reflection of allelic diversity and frequency among the soybean genotypes analyzed were generally high for all the SSR loci tested. PIC values ranged from 0.041 to 0.796, with an average of 0.507. Seven SSR loci revealed PIC values higher than 0.70. Among these, Satt354 and Satt038 are noteworthy due to their relatively high polymorphism (six and five alleles each, respectively), and high PIC values (0.796 and 0.772), respectively. The polymorphism of SSR loci detected in this study was consistent with data obtained in some previous studies (Doldi *et al.*, 1997; Brown-Guedira *et al.*, 2000; Narvel *et al.*, 2000), but was lower than that reported by others (Rongwen *et al.*, 1995; Diwan and Cregan, 1997). The PIC values of our study were in agreement with the data of Doldi *et al.* (1997) and Brown-Guedira *et al.* (2000), who detected mean gene diversity values of 0.50 and 0.69 in a group of 39 and 36 elite/commercial soybean cultivars, respectively.

The five AFLP primer combinations used in this study were selected on the basis of a high number of scorable polymorphic bands. It was possible to discriminate each one of the 44 soybean genotypes using five primer combinations. Band sizes ranged from 100 to 700 bp. The five primer pairs revealed a total of 449 different bands that were of sufficient intensity to be scored, and 208 (46.3%) of these were polymorphic. The percentage of polymorphic bands per assay unit ranged from 34.0% (E-ACT/M-CAT) to 57% (E-AAG/M-CTT), with an average of 46.3%. The average PIC score for AFLP primer combination was 0.12, with a range of 0.08 to 0.16 (Table 3). A similar average PIC score for AFLP was also reported in an earlier study on soybean (Ude *et al.*, 2003). 91 polymorphic bands showed PIC scores > 0.30 indicating that only 20.3% of the 449 bands contributed significantly to the genetic variation of the soybean genotypes. A PIC score > 0.30 has been described previously in soybean based on RFLP (Keim *et al.*, 1992; Lorenzen *et al.*, 1995), RAPD (Thompson and Nelson, 1998) and AFLP (Ude *et al.*, 2003) results and shows its usefulness in other soybean germplasm diversity studies. Thus, the polymorphism seen by SSR and AFLP efficiently distinguished all these accessions of soybean genotypes.

The similarity coefficients based on shared SSR and AFLP bands revealed that the average genetic similarity (GS) between genotypes was 0.446, with a range of 0.220 to 0.765. GS estimates for AFLP and SSR were 0.504 and 0.337, respectively. As expected, the level of polymorphism was higher for SSR (0.507) than for AFLP (0.12), reflecting the hypervariability of SSR markers. SSR/microsatellite analysis thus revealed significantly lower mean genetic similarity values (0.337) than AFLP (0.504). Similar results have been reported for soybean (Powell *et al.*, 1996) and olive (Bandelj *et al.*, 2003). Dendrograms were constructed from genetic similarity data, and clusters were tested for associations. Cophenetic coefficients were fairly high in both molecular systems (SSR = 0.924 and AFLP = 0.958) indicating a good fit for clustering. The Mantel correlation test was used to compare between SSR and AFLP, as well as the combined data. The cophenetic matrix values and the estimated correlations for the two molecular systems and with combination were r = 0.604 (SSR *vs.* AFLP), r = 0.771 (SSR *vs.* combination) and 0.971 (AFLP *vs.* combination), respectively. All these were statistically significant. The slightly lower level of correlation between SSR and AFLP in the present study could probably reflect that these markers are known to target different genomic fractions involving repeat and/or unique sequences, which may have differentially evolved or been preserved during the course of natural or artificial selection.

Cluster analysis based on coefficient of similarity classified the soybean genotypes into four major clusters, which were designated as I, II, III and IV in this study (Figure 1). The dendrogram indicated that 82% of the 44 soybean genotypes clustered in the range of 0.55 to 0.76 similarity coefficients. A correspondence between photoperiodism and place of origin of the cultivars was evident from Figure 1. The Mantel test indicated good fit for the clustering pattern with fairly moderate bootstrap supports (65%-100%). The cluster ‘I' was composed of six genotypes from USA, five from Hungary, three from Philippines, two from Taiwan and one from China, however S-100 appeared as an outlier in this group (Figure 1). Flowering in this group was delayed from 12-68 days in extended photoperiod. Grouping of soybean ancestors/cultivars ftom the USA with Hungarian, French and Japanese genotypes was also reported (Brown-Guedira *et al.*, 2000). The grouping of Jackson, S-100, Evans and Pershing in different subclusters in the present study is in the agreement with previous results (Ude *et al.*, 2003). Cluster II mainly consisted of Indian soybean cultivars (14 cultivars) along with six genotypes from the USA and was again divided into subclusters. The genotypes of this group did not flower under extended photoperiods and are highly photoperiod-sensitive, except for LSb1 and PI424-489A, which flowered after 7 and 14 days under extended photoperiod, respectively. Grouping of six genotypes/cultivars from the USA along with Indian soybean cultivars in II-a is obvious, as most of the initial Indian soybean varieties are either direct introductions from theUSA or were selected or bred using introductions as one of the parent (Karmakar and Bhatnagar, 1996). The genotypes of subgroup II - b comprised only Indian soybean cultivars and clustered together with 53% similarity. The Indian soybean cultivars shown to cluster in this study mainly came from the central and southern zones of India, and the result is in agreement with an earlier report (Hymowitz and Kaizuma, 1981).

Cluster III consisted of four genotypes (three from Hungary (1145/84, Dun NunII-2-15, 1158/84) and one from the USA (Maple Arrow), which grouped together with 0.545 similarities. Though genotype PI 437418 did not group with cluster III, it showed a reasonable level of similarity with this cluster and, thus can be considered as an outlier of this group. The genotypes of this group showed delayed flowering from 1-5 days in extended photoperiod. Cluster IV included one genotype each from Hungary and India. This cluster consisted of diverse genotypes (MACS330, a cross from Monetta (USA) X EC95937 (USSR), and 11/2/1939, a line from Hungary) which showed no delay in flowering under extended photoperiod. The cluster formed by these two genotypes is not strong and showed only 0.50 similarity between each of its members, which, in turn, showed 0.36 similarity with other genotypes of the present study. It is evident from dendrogram (Figure 1) that soybean cultivars/genotypes from the USA grouped along with genotypes of different origin in different clusters, the reason being that a large number of the accessions in the USDA soybean collection are from the same regions of China and Korea. These introductions that make up the base of the American germplasm (Brown-Guedira *et al.*, 2000) were used for development of soybean cultivars in the USA.

Soybean producing regions in India range from the lower Himalayan Hills and Northern Plain in the north to the Deccan Plateau in the south. The soybean varieties cultivated in these areas were developed through separate breeding programs, because most of the Indian soybean varieties are photoperiod sensitive, restricting their cultivation to localized areas only. The genotypes, 11/2/1939 (Hungary) and MACS330 (India) identified as photoperiod insensitive in the present study formed a separate group, as clearly shown by UPGMA (Figure 1). Literature reports indicate that there is a relationship between marker diversity of parents and genetic variance of the resulting progeny. Collecting data on genetic diversity in parents and progeny, however, is time consuming and expensive (Maughan *et al.*, 1996). Thus, identifying genetically diverse parents based for desirable trait based on molecular markers would be a good approach for the production of desirable progeny. This approach has been already used for production of high yielding progeny in soybean (Thompson and Nelson, 1998). In the present study, we are making available potential germplasm resources for photoperiod insensitivity to soybean breeders that can be used for introgression of photoperiod insensitivity genes into soybean cultivars for wider adaptability.

**Figure 1:**
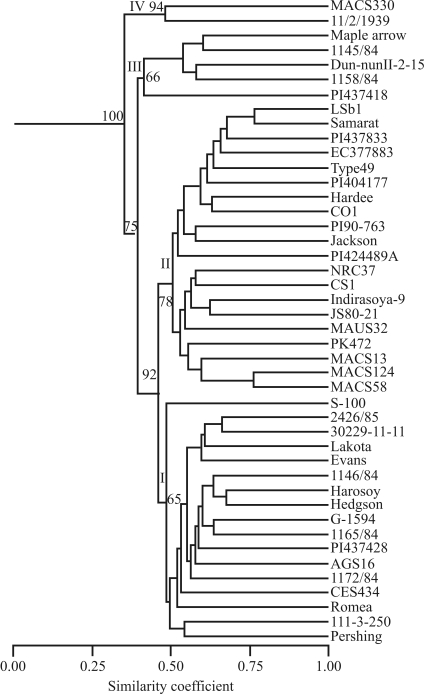
Dendrogram of 44 soybean lines produced by the UPGMA clustering method based on a genetic similarity matrix derived from 120 SSR and 449 AFLP markers. Bootstrap values in percentages for the four major clusters are mentioned at the respective nodes. The major clusters are indicated as I, II, III and IV node on the left side.

## Figures and Tables

**Table 1:** Genotypes and cultivars, country of origin and classification regarding sensitivity to photoperiodism of the 44 soybean genotypes/cultivars used in this study.

S. no.	Collection Id.	Variety or original identity	Country of origin of germplasm collection	Classification*
1	MACS 330	Cultivar	India	I
2	EC 325097	11/2/1939	Hungary	I
3	EC 333897	Maple Arrow	USA	I
4	EC 34101	Dun-NunII-2-15	Hungary	I
5	EC 325118	1158/84	Hungary	I
6	EC 325100	1145/84	Hungary	LS
7	LSb 1	Cultivar	India	LS
8	EC 333922	PI 437418	USA	LS
9	EC 325106	1146/84	Hungary	MS
10	EC 251402	S-100	China	MS
11	EC 333912	PI 424-489A	USA	MS
12	EC 325114	2426/85	Hungary	MS
13	EC 333920	LAKOTA	USA	MS
14	EC 232075	ROMEA	Philippines	MS
15	EC 333880	Evans	USA	MS
16	EC 325159	111-3-250	Hungry	HS
17	EC 325115	1172/84	Hungary	HS
18	EC 333925	PI 437428A	USA	HS
19	EC 325117	1165/84	Hungary	HS
20	EC 333867	Harosoy	USA	HS
21	EC 333919	Hedgson	USA	HS
22	AGS 16	-	Taiwan	HS
23	EC 175321	G-1594	Taiwan	HS
24	EC 242091	30229-11-11	Philippines	HS
25	EC 358004	PI 437833	USA	HS
26	EC 251770	Pershing	USA	HS
27	Samarat	Cultivar	India	HS
28	EC 103336	CES-434	Philippines	HS
29	EC 291448	PI 90-763	USA	HS
30	EC 378783	-	USA	HS
31	EC 333904	PI 404-177	USA	HS
32	EC 251446	Jackson	USA	HS
33	Type 49	Cultivar	India	HS
34	Hardee	Cultivar	India	HS
35	Co1	Cultivar	India	HS
36	JS80-21	Cultivar	India	HS
37	GS1	Cultivar	India	HS
38	NRC 37	Cultivar	India	HS
39	PK 472	Cultivar	India	HS
40	MAUS 32	Cultivar	India	HS
41	Indirasoya-9	Cultivar	India	HS
42	MACS 58	Cultivar	India	HS
43	MACS124	Cultivar	India	HS
44	MACS13	Cultivar	India	HS

*I = Photoperiod insensitive; LS = Low sensitivity; MS = moderate sensitivity; HS = high sensitivity.

**Table 2:** SSR loci, linkage group with position, allele number and polymorphism information content (PIC) for 44 soybean genotypes/cultivars.

SSR primer pair	Primer name	Linkage group	cM position	No. of alleles	PIC
1	Satt276	A1	5.70	3	0.613
2	Satt211	A1	95.96	3	0.369
3	Satt493	A2	35.02	1	0
4	Satt233	A2	100.09	3	0.520
5	Satt415	B1	0.8	5	0.616
6	Satt063	B 2	93.49	4	0.584
7	Satt126	B2	27.63	3	0.644
8	Satt194	C1	26.35	2	0.118
9	Satt524	C1	120.12	1	0
10	Satt170	C2	70.56	2	0.080
11	Satt460	C2	117.77	4	0.575
12	Satt184	Dla	17.52	3	0.581
13	Satt129	Dla	109.67	2	0.384
14	Satt216	D1b	9.80	4	0.728
15	Satt459	D1b	118.6	3	0.224
16	Satt498	D2	32.14	2	0.118
17	Sat_114	D2	84.18	2	0.249
18	Satt231	E	70.23	3	0.503
19	Satt411	E	12.92	6	0.703
20	SOYHSP176	F	68.44	4	0.718
21	Satt072	F	87.01	1	0
22	Satt038	G	1.84	5	0.772
23	Sct_ 187	G	107.11	3	0.249
24	Sat_127	H	28.80	4	0.653
25	Satt434	H	105.74	4	0.663
26	Satt587	I	31.49	1	0
27	Satt354	I	46.22	6	0.796
28	Satt431	J	78.57	4	0.684
29	Sct_046	J	24.09	1	0
30	Satt539	K	1.80	2	0.041
31	SOYPRP1	K	46.94	5	0.752
32	Satt388	L	23.55	1	0
33	Satt278	L	31.22	4	0.639
34	Sat_099	L	78.23	4	0.609
35	GMSC514	M	3.05	3	0.292
36	Satt346	M	112.79	3	0.642
37	Sat_084	N	36.86	2	0.353
38	GMABAB	N	73.10	4	0.749
39	Sat_132	O	8.75	4	0.352
40	Sat_109	O	127.50	5	0.674

**Table 3:** Total number of bands, proportion of polymorphic bands and polymorphism information content (PIC) for each AFLP primer pair used in the analysis of 44 soybean lines.

Primer pair	Total no. of bands	Proportion of polymorphic bands	PIC
E-ACC/M-CAA	89	0.55	0.15
E-AAG/M-CTT	101	0.57	0.16
E-ACC/M-CAC	75	0.47	0.12
E-ACA/M-CAC	86	0.43	0.08
E-ACT/M-CAT	98	0.34	0.097
